# Development of a Patient‐Centered Communication Skills Training: A Qualitative Exploration of Nurse Managers' Perspectives

**DOI:** 10.1002/nop2.70605

**Published:** 2026-05-21

**Authors:** Kendra Mielke, Wiebke Frerichs, Katja Cöllen, Anja Lindig, Martin Härter, Isabelle Scholl

**Affiliations:** ^1^ Department of Medical Psychology University Medical Center Hamburg‐Eppendorf Hamburg Germany

**Keywords:** communication, nurse administrators, patient‐centered care, professional competence, qualitative research

## Abstract

**Background:**

Communication skills training can enhance nursing professionals' patient‐centered communication skills, fostering positive health outcomes for both patients and nursing professionals. Understanding experiences and preferences of the target group is crucial when developing a specific communication skills training. Thus, it is essential to involve not only nurses but also nurse managers in the training development, as they offer a comprehensive overview of communication challenges experienced by nursing staff.

**Aim:**

This study aimed to explore nurse managers' perspectives on essential content for developing a patient‐centered communication skills training.

**Design:**

Qualitative study using semi‐structured interviews.

**Method:**

We conducted interviews with nurse managers working at an academic medical center in Germany. Transcripts of audio‐recorded interviews were analysed using Kuckatz's qualitative content analysis.

**Results:**

*N* = 18 nurse managers from various medical disciplines such as gynaecology, psychiatry, paediatrics, and neurology participated in the study. Participants identified enhancing general communication skills and strategies such as showing empathy and applying tailored communication, addressing communication challenges with patients, fostering professional self‐awareness, communication with relatives, and navigating culturally sensitive communication as potential content of a future patient‐centered communication skills training.

**Conclusion:**

Nurse managers identified several communication aspects which should be included in a communication skills training, with many findings resembling those from international studies on communication in nursing care. However, our results are not limited to specific medical disciplines such as intensive care or oncology. Overall, these findings demonstrate that nurse managers are aware of nurses' communication challenges and needs and are willing to support efforts to improve their communication skills.

**Reporting Method:**

No Patient or Public Contribution.

**Abbreviations**
AL: Anja Lindig
COREQ: consolidated criteria for reporting qualitative research
CST: communication skills training
DM: department manager
DWM: deputy ward manager
IS: Isabelle Scholl
KC: Katja Cöllen
KM: Kendra Mielke
KOMPAT: Communication within patient‐centered nursing care
MH: Martin Härter
NM: nurse manager
UKE: University Medical Center Hamburg‐Eppendorf
WF: Wiebke Frerichs
WM: ward manager

## 1 Background

Patient‐centered communication has been identified as a crucial component of patient‐centeredness (Zill et al. 2015). In clinical nursing practice, patient‐centered communication can be challenging as various tasks, for example, supporting patients' physical and emotional needs, handling administrative tasks, and providing information for relatives often occur simultaneously (Scott et al. 2014).

Communication skills trainings (CST) has been shown to enhance patient‐centered communication in nursing care (Banerjee et al. 2017; Wolderslund et al. 2021). Research indicates participation in a CST can improve self‐efficacy and patient‐centered communication skills (Banerjee et al. 2017; Wolderslund et al. 2021), and is negatively associated with perceived stress and psychological symptoms such as anxiety and depression (Onan et al. 2015). However, current CSTs for nursing professionals in Germany vary in quality and quantity (Jünger and Doll 2016), and are often discipline‐ specific (e.g., oncology). Limited international studies have assessed nursing professionals' communication experiences and needs prior to the development of a corresponding CST (Banerjee et al. 2017; Kerr et al. 2021). Hence, we conducted a focus group study with nurses from various medical disciplines on patient‐centered communication (Mielke et al. 2024). Results showed consistent challenges across disciplines, including dealing with angry or demanding patients and relatives, managing emotions, and providing patient‐centered communication despite organizational barriers. These findings highlight the need to enhance nurses' communication skills (Mielke et al. 2024).

It is essential to consider not only nursing professionals' perspectives but also those of Nurse Managers (NMs) bevor developing a CST, as understanding the entire target group's needs and requirements is crucial (Fernandez et al. 2019). NMs are accountable for creating a safe and supportive work environment for nursing professionals while ensuring patient safety and quality of care (Nurmeksela et al. 2021). They are responsible for the proactive identification of areas necessitating improvement among nursing professionals and providing support through tailored education and training (Kodama and Fukahori 2017; Elsheikh et al. 2023) as well by allocating time for professional development (Bergmann et al. 2024). Considering the significant impact of effective communication on the provision of high‐quality care and patient safety (e.g., by preventing medication errors attributed to communication failures) (Wieke Noviyanti et al. 2021; Timmins 2011), it is imperative that NMs advocate for and facilitate the enhancement of nurses' communication skills (Zuqayl et al. 2024). NMs' own communication skills directly influence nurses' job satisfaction, empowerment, and commitment, as they serve as pivotal role models within the workplace (Fowler et al. 2021).

Despite NMs' importance in fostering effective communication among nurses, there are only a few qualitative international studies that examine NMs' perceptions regarding patient‐centered communication in nursing care (Wittenberg‐Lyles et al. 2013; Sawin et al. 2019). Two different qualitative studies highlighted communication barriers faced by oncology nurses, including communication inconsistencies and interprofessional difficulties, and emphasized the importance for post‐graduate training to improve patient‐centered communication (Wittenberg‐Lyles et al. 2013; Sawin et al. 2019). Since one major strength of qualitative research is its ability to provide a deeper understanding of various patterns and structures within a specific real‐world problem or phenomenon, conducting qualitative studies is essential for exploring the underlying mechanisms (Tenny et al. 2025). Given the important role NMs play in facilitating patient‐centered communication among their staff, it is essential to include their perspectives and attitudes on this topic.

To our knowledge, no studies have specifically investigated the perspectives of German NMs from various medical disciplines regarding required content for a CST. Therefore, the aim of this study was to conduct interviews with NMs in Germany to gather their perspectives on essential content for a patient‐centered CST, thereby facilitating the development of such a training.

## 2 Method

### 2.1 Design

To develop a patient‐centered CST that is tailored to the nursing professionals' needs, we carried out interviews with a semi‐structured interview guide. We selected this qualitative methodology as it facilitates an in‐depth understanding of individual perspectives on specific research topics. In preparation of this manuscript, we followed the consolidated criteria for reporting qualitative research (COREQ, see File S1) (Tong et al. 2007).

### 2.2 Study Setting and Recruitment

The present study is part of the KOMPAT study (German: KOMmunikation in der PATient:innenorientierten Pflege; English: Communication within patient‐centered nursing care), which was designed to develop and evaluate a patient‐centered CST for nursing professionals (Lindig et al. 2024), conducted at University Medical Center Hamburg‐Eppendorf (UKE), Germany. In September 2021, we informed department managers (DMs) of all 13 departments of the UKE about the KOMPAT study and the present study's background and objectives. Subsequently, invitation e‐mails for *n* = 9 DMs and *n* = 84 ward managers (WMs) and deputy ward managers (DWMs) were dispatched between December 2021 and January 2022 (in the following text, the term NMs includes DMs, WMs, and DWM). For detailed information on job titles, see Table 1. Interested NMs could apply to participate in an interview by contacting the study team via email or telephone. We determined a priori that 15–20 participants should be interviewed, as recommended in literature (Moser and Korstjens 2018). Further, we employed purposive sampling, selecting participants based on researchers' assumptions of who would be most likely to provide valuable information for addressing the research (Moser and Korstjens 2018; Palinkas et al. 2015). Using this approach, we aimed for high variability in management positions (i.e., DMs, WMs, DWMs) and medical disciplines (i.e., NMs from different departments). During the sampling process, we collected information on these two criteria and approached or re‐invited NMs from positions or medical disciplines not yet interviewed. However, due to limited resources faced by NMs and low response rate from some departments, we were unable to cover all medical disciplines.

**TABLE 1:** Areas of responsibility and tasks of nurse managers (information based on UKE employees' reports).

Job title	Area of responsibility	Tasks
DM—Department manager	Superior management and coordination of all wards within one department in the hospital	Strategic planning, quality management, staff leadership and training, collaboration with hospital management No involvement in direct nursing care
WM—Ward manager	Superior management of a ward within the hospital	Duty planning, direct staff management, work process organization, acting as a contact person for patients and relatives, cooperation with physicians and other disciplines Depending on ward, little or no involvement in direct nursing care
DWM—Deputy ward manager	Support and deputise for the ward manager	Carrying out management tasks in the absence of the ward manager, support with administrative tasks (e.g., duty planning) Depending on the ward, partial to full involvement in direct nursing care

We decided to apply data saturation, as this approach is common for content analysis (Rahimi and Khatooni 2024; Saunders et al. 2018). Data saturation occurs during data collection or analysis when additional data no longer contribute to answer the research question (Rahimi and Khatooni 2024; Saunders et al. 2018). After each interview, two members of the research team (KM and KC) debriefed and discussed its content to identify new or redundant aspects. Based on this, we determined data saturation prior to category development. Due to the study's strict time frame, we did not monitor saturation continuously during analysis, as we had no opportunity to conduct further interviews if needed.

### 2.3 Inclusion and/or Exclusion Criteria

Participants of the semi‐structured interviews needed to hold a management position as DM, WM, or DWM within one of the UKE's departments. Nursing professionals in non‐management positions were not permitted to participate, as they were involved in another study which results are reported elsewhere (Mielke et al. 2024).

### 2.4 Data Collection

Interviews were conducted in January and February 2022. Either KC or KM conducted the interviews. Each interview was audio‐recorded. Due to the ongoing COVID‐19 pandemic, participants were given the option to be interviewed online via a meeting platform or in person at the research team's department or their own office. Interview appointments were scheduled individually. No prior relationships existed between interviewers and participants.

An interview guide (see Table 2 and File S2) was developed and discussed with researchers experienced in conducting interview studies (AL, MH, IS), as well as pilot‐tested with two nurses in management positions not affiliated with the UKE. Before starting the interview, the respective interviewer (KM or KC) introduced herself, explained the study's purpose, highlighted the importance of the interviewee's perspective on the topic, and outlined the agenda for the interview. Additionally, we collected demographic data through a brief questionnaire. Participants were not invited to comment or correct the transcript, nor did they receive the results to comment on them.

**TABLE 2:** Interview guide for semi‐structured interviews.

Interview guide
A. Start Introduction of interviewer (name, profession, work experience) Explanation of the interview's purpose and the agenda B. Training content In your opinion, what content should be included in a training on patient‐centered communication skills? If there is no more response or if questions arise about the specific meaning of the question, the interviewer can show a list of topics known from previous studies (see File S2) Please name three core topics out of the mentioned topics that you think should definitely be included in a training‐ So what would be number 1, 2, and 3 for you? Why are these topics particularly important? C. Closing Additional remarks Inquiries for the interviewer Appreciation and closure

### 2.5 Data Analysis

For this study, we analysed only responses related to NMs' perspective on nurses' needs and requirements regarding the content for a patient‐centered CST. Therefore, responses concerning participants' opinion on organizational aspects of training development were not included in the analysis.

Two student assistants transcribed all audio files with the transcription software F4 (version 4.2, dr.dresing & pehl GmbH, Marburg, Germany) by following the transcription rules of Dresing & Pehl (Dresing and Pehl 2015). To minimize potential misunderstandings during transcription, one member of the study team (either KC, WF, or KM) thoroughly reviewed all transcripts. During transcription, personal data (e.g., names of colleagues or superiors, patient‐related information) was pseudonymized. The pseudonymization list was not digitalized and was kept as a paper document in a lockable cabinet in WF's office.

Data analysis was conducted by KM and KC and supervised by WF, in accordance with the principles of Kuckatz's content analysis (Kuckartz 2018), utilizing MAXQDA software (VERBI GmbH, Berlin, Germany). First, KC and KM developed deductive main categories based on topics known form existing research (Wittenberg‐Lyles et al. 2013; Aslakson et al. 2012; Banerjee et al. 2016) and discussed it with WF. KC and KM each double coded 20% of the transcripts (4 out of 17 transcripts) to ensure reliability. The remaining 13 transcripts were allocated between KC and KM for individual coding. When new aspects emerged, KC and KM discussed and incorporated them as inductive main categories. Additionally, inductive sub‐categories were created for detailed differentiation within a main category, if necessary, gradually building a preliminary category system. In a final stage, KM applied the preliminary category system to code the entire dataset again, making final adjustments to establish the final category system. Throughout all steps, WF was consulted as needed.

### 2.6 Rigour and Reflexivity

All authors have previous experiences in collecting qualitative data through semi‐structured interviews as well as evaluating qualitative data applying content analysis. KM is a female health scientist (M.A.), trained physical therapist and a doctoral researcher. WF is a female health scientist (M.Sc.), international physical therapist (B.Sc.), and experienced doctoral researcher. KC is a female psychologist (M.Sc.) and a trained nurse. AL is a female neuro‐psychologist (M.Sc.), trained psycho‐oncologist, and post‐doctoral researcher. MH and IS supervised the trial as principal investigators. MH is a male physician, psychologist, and professor for medical psychology. IS is a female psychologist, and professor for psycho‐oncology.

### 2.7 Ethical Considerations

The study was approved by the Local Psychological Ethical Committee of the Center for Psychosocial Medicine at the University Medical Center Hamburg‐Eppendorf (approval number LPEK‐0367). We followed the most recent version of the World Medical Association's Helsinki Declaration and applied the rules of ethical scientific conduct. Requirements for data protection were met. Participants were informed about the study objective and data protection and had to give their written consent by signing a consent form beforehand. Participation was voluntary.

## 3 Results

### 3.1 Sample Characteristics

Overall, we conducted *n* = 17 interviews with *n* = 18 NMs, as two WMs asked to be interviewed together. Of these, *n* = 7 were DMs, *n* = 8 were WMs and *n* = 2 were DWMs. *N* = 63 WMs and DWMs did not respond to the invitation e‐mail. Additionally, *n* = 10 interested WMs and DWMs agreed to participate, which we declined, as we had already achieved data saturation by interviewing NMs across various medical disciplines and with a broad range of work experience. Two participants preferred face‐to‐face interviews; all other interviews were conducted online using the meeting platform Webex. The interviews lasted between 25 and 58 min (mean 39 min) and were audio‐recorded. Most of the 18 participating NMs were female (*n* = 13) and had more than 10 years of professional experience in nursing care (*n* = 14). Participants came from diverse medical disciplines, including gynaecology, obstetrics, intensive care, psychiatry, paediatrics, cardiology, oncology and neurology. The number of participants from each discipline is not described to prevent identification. For more information on participants' characteristics, see Table 3.

**TABLE 3:** Participants' characteristics (*n* = 18).

	*n* (%)
Gender	
Female	13 (72%)
Male	5 (28%)
Age	
< 30 years	4 (22%)
31–40 years	7 (39%)
41–50 years	3 (17%)
> 50 years	4 (22%)
Position	
Department manager	7 (39%)
Ward manager	8 (44%)
Deputy ward manager	3 (17%)
Professional experience in nursing care	
< 5 years	1 (6%)
5–10 years	2 (11%)
11–20 years	7 (39%)
> 20 years	7 (39%)
Not disclosed	1 (7%)

### 3.2 Participants' Perspective on Patient‐Centered Communication Skills

Based on the aim of this study, six deductive main categories and respective inductive sub‐categories emerged from the content analysis: 1. Communication skills and strategies; 2. Communication challenges with patients; 3. Professional self‐awareness; 4. Communication with relatives; 5. Culturally sensitive communication; and 6. Handling time constraints. Additional sub‐categories were identified within these main categories. Representative quotes for each category are provided in Table 4.

**TABLE 4:** Main and sub‐categories with exemplar quotes.

Main category 1: Communication skills and strategies	
Sub‐category 1.1: Knowledge about and application of communication theory	‘Well, the content probably [should include] general communication theory that we all have already gone through in our vocational training. […] I would definitely include it as a refresher, because these are fundamental theories […]’. (Interview DWM 06)
Sub‐category 1.2: Non‐verbal communication	‘Especially nonverbal communication, when you are under a lot of stress and don't look up, […] a patient is standing in front of the registration desk and sometimes it is enough to just look up and nod, like “I see you”’. (Interview DWM 06)
Sub‐category 1.3: Appropriate language	‘[…] I don't want to hear ‘don't get scared’ anymore, I am automatically scared if someone says that and that I am afraid. […] And that is missing somehow, the comprehension of nursing staff’. (Interview DWM 04)
Sub‐category 1.4: Showing Empathy	‘Well, […] for me, that is kind of a door opener, you have to have this empathy, […] that is also an important story’. (Interview WM 10)
Sub‐category 1.5: Building relationship	‘I have to build a relationship with the patient, my counterpart. That is essential and how I manage that varies, and some people [nurses] fail to do so’ (Interview WM 10)
Sub‐category 1.6: Tailoring Communication	‘And to translate, […] that they can translate this well for patients and that they can adapt to different counterparts of course. […] In other areas, there are always difficulties, so you have to repeat a lot and communicate according to the intended audience. I think that is a very, very important topic’. (Interview DM 03)
Sub‐category 1.7: Conversation structure	‘Well, I think it is really about a good entrée [in a conversation]. So, when someone arrives, to pick them up from where they are. […] What is the person's emotional set‐up and what is their actual problem’. (Interview WM 01)
Main category 2: Communication challenges with patients	
Sub‐category 2.1: Discrimination based on cultural background and sexual harassment	‘But we have also received feedback that some nurses with another cultural background have experienced discrimination from patients. And then the question arises how to deal with that in communication’. (Interview DM 12)
Sub‐category 2.2: Patients with cognitive impairments or physical limitations affecting communication	‘But generally challenging patient situations arise when they [patients] have cognitive impairments and either as a result of their illness or their therapy are not so accessible, and yet [one] still have to find a way. […] And in daily work, it often happens that one becomes rather unfriendly and tends to abruptly cut off, which usually does not make the situation any better’ (Interview DM 07)
Sub‐category 2.3: Aggressive or demanding patients	‘So how can I deal with very demanding behavior of patients, how should I react. Kind of de‐escalating communication, I think that is actually really important’. (Interview DM 03)
Sub‐category 2.4: Patients with serious illness, dying patients	‘And in our field, this has become more and more important‐ communication with cancer patients. Meaning, that a different approach is needed, that sensitive topics have to be discussed. Topics that patients may not want to deal with at all’. (Interview WM 11).
Main category 3: Professional self‐awareness	
Sub‐category 3.1: Establishing professional patient–nurse relationship	‘And topics of closeness and distance and also authenticity and then how can I, as a nurse, distance myself or stop a conversation. […] How it is possible on the ward. I think that should definitely be a focus’. (Interview DWM 13).
Sub‐category 3.2: Self‐reflection	‘Then also, I think, the topic of mindfulness. How am I here today? […] How can I deal with it? One possibility is: I keep to myself and say nothing or I deal with it openly and admit to myself that today is not my day’. (Interview WM 02)
Main category 4: Communication with relatives	‘Then I think it would be very, very important‐ as I am convinced that this is an increasingly important resource for us as nursing staff in the context of the treatment team – to involve the relatives’. (Interview WM 02)
Main category 5: Culturally sensitive communication	‘And probably, you are right; we have to look at the cultural aspects […]. So that would probably also be useful in communication skills training’. (Interview WM 07)
Main category 6: Handling time constrains	‘I think, communication in stressful situations is a topic that is becoming increasingly important’. (Interview DM 09)

#### 3.2.1 Main Category 1: Communication Skills and Strategies

When exploring which general communication skills and strategies would be beneficial for enhancing nurses' patient‐centered communication, participants indicated that knowledge about and application of communication theory, non‐verbal communication, the use of appropriate language, as well as skills in showing empathy, building relationships with patients, tailoring communication, and structuring conversations should be prioritized.

##### 3.2.1.1 Sub‐Category 1.1: Knowledge About and Application of Communication Theory

Participants believed it would be beneficial to refresh nurses' knowledge of well‐known communication theories, such as Nonviolent Communication (developed by Marshall Rosenberg) and key elements of Carl Rogers' person‐centered therapy. Furthermore, they also considered that discussing skills derived from pedagogy could be helpful.

##### 3.2.1.2 Sub‐Category 1.2: Non‐Verbal Communication

The significance of nonverbal communication was highlighted. Participants pointed out that even small gestures, such as a nod of the head, can convey empathy and help patients feel recognized. They found it crucial for nursing professionals to recognize the pivotal role nonverbal communication has in interactions with patients.

##### 3.2.1.3 Sub‐Category 1.3: Appropriate Language

Appropriate language was considered relevant to provide patient‐centered communication. In participants' opinion this included addressing patients appropriately, using concise sentences, avoiding technical jargon, and employing culturally sensitive language. Additionally, it was noted that nursing professionals should enhance their awareness of the potential negative impact of using fear‐inducing statements. This includes emphasizing only patients' deficits and issuing stringent directives about the behaviours or movements that patients are required to follow or avoid.

##### 3.2.1.4 Sub‐Category 1.4: Showing Empathy

Participating NMs highlighted the importance of displaying empathy when communicating with patients. They emphasized that future CSTs should focus on understanding the patients' perspectives, necessitating an individualized approach that is tailored to the specific needs of patients.

##### 3.2.1.5 Sub‐Category 1.5: Building Relationship

Participants stated that building relationships with patients is crucial. They mentioned that building strong relationships with patients prevents misunderstandings and facilitates the management of highly emotional situations. Further, employing appreciative communication to build robust relationships and ensure patients feel understood was deemed essential. To foster patients' trust, skills like taking time, sitting down on the bedside, and active listening were considered advantageous and should be incorporated in a CST. Moreover, NMs mentioned that respecting patients' needs can enhance satisfaction for both patients and nurses. Participants emphasized the necessity of integrating patients into care processes, particularly when more than one nurse is involved in providing care. Additionally, the importance of permitting emotional and physical closeness within a professional setting was mentioned.

##### 3.2.1.6 Sub‐Category 1.6: Tailoring Communication

Delivering communication tailored to the individual patients' needs was perceived as essential. This included considering patients' psychosocial characteristics (e.g., cultural background, age) and providing adapted information accordingly. Other topics mentioned were verifying patients' understanding, offering clear step‐by‐step explanations to foster patients' self‐management, and disseminating information about specific situations (e.g., explaining the necessity of certain treatments or post‐operative movement limitations).

##### 3.2.1.7 Sub‐Category 1.7: Conversation Structure

Interviewed NMs mentioned that participants of a CST would benefit from acquiring skills in conversation structuring. It was emphasized that the initial phase of a conversation would entail managing conversation initiation as well as selecting an appropriate setting. Participants stated that conversation guidelines could assist nurses in organizing recurrent situations (e.g., medical history inquiries, hospital admissions, and discharges), potentially enhancing the exchange of necessary information. Furthermore, participants highlighted the importance of learning standardized skills for structuring conflict communication and breaking bad news. Additionally, standardizing specific conversations could promote consistency among all parties involved.

#### 3.2.2 Main Category 2: Communication Challenges With Patients

When investigating participants' views on communication challenges, NMs highlighted that nurses frequently encounter highly challenging situations with patients in which communication skills could serve as a valuable tool. They described situations involving discrimination and sexual harassment that occur. Additionally, communication challenges arise when working with cognitively impaired or physically limited patients, as well as in situations where patients exhibit aggressive or demanding behaviour. Communication with patients who are seriously or terminally ill was also mentioned as a challenge.

##### 3.2.2.1 Sub‐Category 2.1: Discrimination Based on Cultural Background and Sexual Harassment

NMs considered it important for nursing professionals to acquire skills to respond professionally yet assertively to instances of discrimination. An example of workplace discrimination was recounted, involving racist comments directed at a medical assistant wearing a headscarf. Another aspect mentioned was that responding to inappropriate patient behaviour, including sexual innuendo, poses a significant challenge for nursing professionals and should therefore be incorporated in a CST.

##### 3.2.2.2 Sub‐Category 2.2: Patients With Cognitive Impairments or Physical Limitations Affecting Communication

Participants highlighted that nursing professionals often struggle to communicate with patients who have cognitive impairments or physical limitations affecting their communication abilities. They emphasized the need for nursing professionals to have assistance and essential training to effectively interact with patients affected by conditions such as dementia or delirium, as well as those unable to verbally communicate due to treatment effects (e.g., tracheal intubation).

##### 3.2.2.3 Sub‐Category 2.3: Aggressive or Demanding Patients

A frequently discussed aspect was the critical role of communication in de‐escalating situations involving aggressive or demanding patients. Participants highlighted the importance of effective communication in this context. It was reported that some nurses experienced significant fear due to verbal threats from angry patients. According to participants, many nurses found it challenging to manage dissatisfied and noisy patients. Further challenges arise from dealing with patients who are frequently upset and complain, those who are agitated and demanding, or impatient patients. Attention was drawn to the aspect that a CST should also emphasize the importance of nurses being aware of available support resources in such situations.

##### 3.2.2.4 Sub‐Category 2.4: Patients With Serious Illness, Dying Patients

Participants suggested that communication with patients with serious illnesses as well as communication about dying and death should be included in a CST. Several specific challenges were identified in this context: Finding the right words in such situations, communicating with patients after they have received a bad or life‐changing diagnosis, supporting patients following such diagnosis, breaking bad news, and communicating with patients in different stages of their illness (e.g., terminal stage). Other participants highlighted the relevance of understanding the process of dying for effective communication, as well as the necessity of ethical perspectives when communicating about death and dying.

#### 3.2.3 Main Category 3: Professional Self‐Awareness

When exploring the importance of professional self‐awareness, participants emphasized the significance of maintaining personal boundaries and managing emotions. Therefore, they highlighted the need for specific communication skills to foster a professional relationship with the patient, as well as the importance of reflecting on one's own boundaries and insecurities and communicating them appropriately.

##### 3.2.3.1 Sub‐Category 3.1: Establishing Professional Patient–Nurse Relationship

Participating NMs identified maintaining emotional balance (e.g., avoiding excessive emotional involvement) as one key element for patient‐centered communication. Nurses attending a CST should learn strategies and skills to manage their own emotions effectively. This includes sharing positive emotions (e.g., joy) without compromising professionalism and finding a balance between closeness and distance in patient interactions. Additionally, strategies should be discussed on how not to take patient frustration personally or feel offended, as well as how to be aware of and utilize personal resources to mediate challenging situations.

##### 3.2.3.2 Sub‐Category 3.2: Self‐Reflection

For participating NMs, self‐awareness encompasses nurses recognizing their mental state and personal limits, and learning to establish boundaries accordingly. It was considered crucial for nurses to acknowledge these limits without embarrassment and identify when to consult other professionals (e.g., psychologists) or seek support for themselves (e.g., managing challenging situations). The first step in setting boundaries is learning to recognize and reflect about inner conflicts (e.g., difficulties in discussing death and dying due to personal experiences with the death of a family member). Additional aspects mentioned include managing personal insecurity, reflecting on and expressing personal emotions and energy levels.

#### 3.2.4 Main Category 4: Communication With Relatives

Participants frequently referred to communication with relatives and identified this as an important aspect which should be integrated into a CST, as this topic requires enhanced communication skills. They particularly emphasized the challenges of interacting with demanding relatives (e.g., those who are angry or hot‐tempered). Furthermore, they noted the complexities of communicating with relatives from diverse cultural backgrounds, highlighting varying behaviours toward ill family members. The importance of engaging with relatives of patients receiving palliative care was also underscored. One participant indicated that in certain situations (i.e., patient is in a comatose state), verbal communication with patients is no longer possible, thereby positioning relatives as the primary contacts for decision‐making. Hence, communication with relatives was perceived as a substantial resource alongside patient communication.

#### 3.2.5 Main Category 5: Culturally Sensitive Communication

When analysing participants' views on culturally sensitive communication, NMs emphasized the importance of developing skills to effectively address language and cultural differences. Further, NMs noted that increased cultural diversity among patients and nursing professionals sometimes results in communication challenges due to language barriers. As behaviours could be influenced by a person's cultural background, understandings of intimacy and personal boundaries might also arise. According to NMs, this divergence among individuals from various cultural backgrounds could lead to misunderstandings, affecting communication. One participant highlighted the significance of raising awareness of cultural diversity, as it fosters acceptance and tolerance.

#### 3.2.6 Main Category 6: Handling Time Constrains

When examining the impact of time constraints on nurses' daily routines, participants emphasized that improving communication skills is also essential under these circumstances, as maintaining professionalism while simultaneously demonstrating empathy toward patients remains important. Further, it was considered essential for nursing professionals to foster the capacity to retain a comprehensive overview in stressful situations and focus on essential aspects of care (e.g., by prioritizing patient needs). Concurrently, nurses should be encouraged to develop strategies that facilitate the maintenance of composure and the provision of thoughtful responses in these challenging circumstances.

### 3.3 Prioritization of Aspects for a CST

After participants shared their insights regarding relevant content for a CST, we asked them to prioritize their top three aspects from all their mentioned aspects. Subsequently, the interviewer summarized all aspects discussed in the respective interview before the NM were asked to prioritize. Out of 18 participants, 14 responded to this question.

The majority of interviewed NMs prioritized the following aspects as essential content for a CST: (a) learning to apply general communication skills and strategies—particularly for building relationships, conveying empathy and providing tailored communication—and (b) managing challenging situations, with a focus on de‐escalation skills for interactions with demanding or angry patients, as well as discussing serious illness, death and dying. Fewer participants identified (c) communication with relatives, (d) professional self‐awareness, and (e) culturally sensitive communication as most relevant for a CST.

## 4 Discussion

The present study explored NMs' perspective concerning essential content for a patient‐centered CST. The findings revealed that NMs identified enhancing communication skills and strategies, navigating communication in challenging situations involving patients and their relatives, as well as fostering professional self‐awareness as important elements to be incorporated into a CST.

The training needs identified in this study align with the results of the focus group study with nurses, also conducted by the research team (Mielke et al. 2024). It was identified in both studies that managing challenging situations with demanding or aggressive patients and discussing serious illness and dying patients are especially challenging and essential to include in a CST. Both groups emphasized the importance of general communication skills and strategies, communication with relatives, and cultural aspects (Mielke et al. 2024). These overlaps might be explained by the fact that all ward and deputy ward managers have previously worked, or in some cases still work, as nurses on wards and are therefore familiar with communication challenges that arise in clinical practice. Further, as nurse managers maintain close contact with their nursing teams, they have firsthand knowledge of the daily challenges faced in patient care.

While focus group participants identified managing emotions as challenging, they did not prioritize it for a CST. However, NMs emphasized professional self‐awareness, indicating they are more likely to focus on nurses' mental well‐being than nurses themselves. This increased focus may arise from NMs' attempt to assess their ward or department from a broader perspective when evaluating processes (Kodama and Fukahori 2017), considering not only organizational factors but also individual aspects of nurses. Simultaneously, they are trained to be aware of nurses' well‐being and to proactively advocate for it (Kodama and Fukahori 2017). Self‐awareness is a dynamic and iterative process of reflecting on one's own characteristics, emotions, beliefs, and actions (Hamaideh et al. 2024). Literature indicates that enhancing nurses' self‐awareness improves communication skills and self‐efficacy, supports recognition of personal stress symptoms, serves as a preventive tool against burnout, and fosters the establishment of professional relationships and the development of empathy (Rasheed et al. 2019). Further, a study by Younas et al. (2020) suggests that enhancing nurses' self‐awareness may also facilitate the improvement of cultural competencies, sensitizing nurses to the needs of patients with diverse cultural backgrounds. However, most CST do not focus on self‐awareness (Denniston et al. 2017). Additionally, comprehensive studies detailing these benefits, particularly from patients' perspectives, remain lacking (Rasheed et al. 2019).

The highlighted importance of fostering nurses' general communication skills and strategies such as showing empathy and applying non‐verbal communication is also in line with a focus group study with NMs working in oncology, conducted by Wittenberg‐Lyles et al. (2013). Participants of that study emphasized the use of tailored information and appropriate communication about pain and spirituality (Wittenberg‐Lyles et al. 2013).

There is existing evidence on effective methods for teaching communication skills and strategies to nurses, such as providing feedback, enabling self‐reflection, and offering follow‐up sessions (Ammentorp et al. 2022; Byrne et al. 2024). However, since communication involves not only learned skills but also learners' attitudes and personality, changing nurses' communication behaviour remains challenging (Grant et al. 2025). Therefore, it is essential to recognize the complex nature of communication and adopt a holistic approach when teaching these skills (Grant et al. 2025).

Another aspect mentioned by participating NMs was the importance of learning more about patient‐centered communication in challenging situations. Communication with demanding or aggressive patients as well as relatives was specifically described as highly challenging. Research indicates a direct link between patients' aggressive communication and nurses' health outcomes, as well as an indirect link through communication satisfaction (Lam 2023). Considering this, it becomes even more important to equip nurses with communication skills and strategies to manage aggressive communication, thereby fostering communication satisfaction and mitigating negative health outcomes. Similarly, to NMs in this study, interviewed paediatric oncology NMs in a study by Sawin et al. (2019), observed a lack of general communication skills, particularly in palliative and end‐of‐life communication, in early career nurses. They indicated that the current approach is more about learning by doing rather than receiving formal education in communication (Sawin et al. 2019). To foster nurses' palliative and end‐of‐life communication skills, appropriate training is beneficial (Fuoto and Turner 2019).

The study possesses significant strengths. We successfully recruited NMs from diverse medical disciplines, thus encompassing a broad spectrum of perspectives and experiences. Although NMs are not directly or only occasionally involved in clinical care activities, their supervisory roles afford them a comprehensive overview of the communication challenges in nurses' daily work. Therefore, their insights are broadly applicable and hold considerable value for the development of patient‐centered CSTs.

Due to the selected methodology, several limitations must be acknowledged. Due to their areas of responsibility, DMs' and WMs' perspectives on communication challenges in nursing care are often informed by their previous experiences or second‐hand information obtained from their staff, as not all NMs are currently engaged in direct care. This reliance on indirect information may potentially result in the omission of certain critical aspects. Additionally, after NMs could not recall further content for a future CST, they were provided with a list of communication challenges and scenarios from previous CST studies and asked if these should also be included as content. This may have influenced their responses. The study exclusively involved NMs from a single academic medical center, which can potentially limit the applicability of the results to NMs working in other hospitals, settings, and regions.

## 5 Conclusion

The NMs involved in the present study identified and prioritized various aspects to be included in a future patient‐centered CST. Several identified aspects, including the enhancement of general communication skills and strategies and improving end‐of‐life communication, are consistent with findings from international studies examining nursing professionals' perspectives on communication challenges within nursing care. Our findings indicate that these challenges are not confined to oncology and intensive care but extend to other medical disciplines such as gynaecology, obstetrics, psychiatry, paediatrics, cardiology, and neurology. Additionally, the present findings indicate that NMs exhibit a strong emphasis on nurses' mental well‐being. They acknowledge the importance of patient‐centered communication within nursing practice and are prepared to facilitate the development of nurses' communication skills.

The insights garnered from this study, along with results from focus groups with nurses (Mielke et al. 2024), were included in the development of a patient‐centered CST, which focuses on applying general communication skills, de‐escalation communication, and communication about serious illnesses, death, and dying. The CST is currently undergoing evaluation in a randomized controlled trial (Lindig et al. 2024).

## Author Contributions

**Kendra Mielke:** conceptualization, methodology, validation, formal analysis, investigation, data curation, visualization, writing – original draft. **Wiebke Frerichs:** conceptualization, methodology, validation, formal analysis, data curation, project administration, writing – review and editing. **Katja Cöllen:** conceptualization, methodology, validation, formal analysis, investigation, data curation, writing – review and editing. **Anja Lindig:** conceptualization, methodology, writing – review and editing. **Martin Härter:** conceptualization, methodology, validating, funding acquisition, supervision, writing – review and editing. **Isabelle Scholl:** conceptualization, methodology, validating, funding acquisition, supervision, writing – review and editing.

## Funding

This study is funded by the German health insurance fund DAK‐Gesundheit. The funding body was not involved in the design of the study, collection, analysis, and interpretation of data nor in writing the manuscript. We acknowledge financial support from the Open Access Publication Fund of UKE ‐ Universitätsklinikum Hamburg‐Eppendorf.

## Ethics Statement

The study was approved by the Local Psychological Ethical Committee of the Center for Psychosocial Medicine at the University Medical Center Hamburg‐Eppendorf (approval number LPEK‐0367). We followed the most recent version of the World Medical Association's Helsinki Declaration and applied the rules of ethical scientific conduct. Requirements for data protection were met.

## Consent

Participants were informed about the study objective and data protection and had to give their consent by signing a consent form beforehand. Participation was voluntary.

## Conflicts of Interest

K.M., A.L., and K.C. declare that they have no competing interests. I.S. and M.H. declare that they currently are (M.H.) or have been (I.S.) members of the executive board of the International Shared Decision Making Society, which has the mission to foster shared decision making implementation and patient‐centeredness. W.F. is currently an active member of tEACH, the teaching subcommittee of the International Association for Communication in Healthcare (EACH), which has the mission to explore and improve communication in healthcare. M.H., W.F., and I.S. have no further competing interests.

## Supporting information


**File S1:** COREQ checklist.

**File S2:** List of possible aspects to include in a communication skills training.

## Data Availability

The datasets used and analysed during the current study are not publicly available due to their close association with participants' working area, making it difficult to sufficiently anonymize all personal data in all documents. Deidentified data (in German language) that support the findings of this study are available on reasonable request. Investigators who propose to use the data have to provide a methodologically sound proposal directed to the principal investigator of the study (Prof. Dr. Isabelle Scholl). Signing a data use/sharing agreement will be necessary, and data security regulations both in Germany and in the country of the investigator who proposes to use the data must be complied with. Preparing the data set for use by other investigators requires work and is thus linked to available or provided resources.
