# Supplementary material for: Development of a Patient‐Centered Communication Skills Training: A Qualitative Exploration of Nurse Managers' Perspectives
Source: Nurs Open. 2026 May 21;13(5):e70605. doi: 10.1002/nop2.70605 (PMC13240314; doi:10.1002/nop2.70605)
Supplement: Supplementary file 1 — File S1: COREQ checklist. [file NOP2-13-e70605-s001.doc]

**Supplementary file 1: COREQ (COnsolidated criteria for REporting Qualitative research) Checklist**

| Topic | ItemNo | Guide Questions/Description | Reported on page No. |
| --- | --- | --- | --- |
| **Domain 1: Research team and reflexivity** | | | |
| Personal characteristics | | | |
| Interviewer/facilitator | 1 | Which author/s conducted the interview or focus group? | See page 5 and 6, section “Data collection” |
| Credentials | 2 | What were the researcher’s credentials? E.g. PhD, MD | See page 7, section “Rigor and reflexivity” |
| Occupation | 3 | What was their occupation at the time of the study? | See page 7, section “Rigor and reflexivity” |
| Gender | 4 | Was the researcher male or female? | See page 7, section “Rigor and reflexivity” |
| Experience and training | 5 | What experience or training did the researcher have? | See page 7, section “Rigor and reflexivity” |
| Relationship with participants | | | |
| Relationship with participants | 6 | Was a relationship established prior to study commencement? | See page 5 and 6, section “Data collection” |
| Participant knowledge of the interviewer | 7 | What did the participants know about the researcher? e.g. personal goals, reasons for doing the research | See page 6, section “Data collection” (table 2 “Interview guide for semi-structured interviews” |
| Interviewer characteristics | 8 | What characteristics were reported about the interviewer/facilitator? e.g. Bias, assumptions, reasons and interests in the research topic | See page 7, section “Rigor and reflexivity” |
| **Domain 2: Study design** | | | |
| Theoretical framework | | | |
| Methodological orientation and Theory | 9 | What methodological orientation was stated to underpin the study? e.g. grounded theory, discourse analysis, ethnography, phenomenology, content analysis | See page 6 and 7, section “Data analysis” |
| Participant selection | | | |
| Sampling | 10 | How were participants selected? e.g. purposive, convenience, consecutive, snowball | See page 4 and 5, section “Study Setting and Recruitment” |
| Method of approach | 11 | How were participants approached? e.g. face-to-face, telephone, mail, email | See page 4 and 5, section “Study Setting and Recruitment” |
| Sample size | 12 | How many participants were in the study? | See page 8, section “Sample characteristics” |
| Non-participation | 13 | How many people refused to participate or dropped out? Reasons? | See page 8, section “Sample characteristics” |
| Setting | | | |
| Setting of data collection | 14 | Where was the data collected? e.g. home, clinic, workplace | See page 5 and 6, section “Data collection” |
| Presence of non-participants | 15 | Was anyone else present besides the participants and researchers? | See page 5 and 6, section “Data collection” |
| Description of sample | 16 | What are the important characteristics of the sample? e.g. demographic data, date | See page 8, section “Sample characteristics” and table 3 “Participants characteristics” |
| Data collection | | | |
| Interview guide | 17 | Were questions, prompts, guides provided by the authors? Was it pilot tested? | See page 5 and 6, section “Data collection” (table 2 “Interview guide for semi-structured interviews” |
| Repeat interviews | 18 | Were repeat interviews carried out? If yes, how many? | Not applicable |
| Audio / visual recording | 19 | Did the research use audio or visual recording to collect the data? | See page 5 and 6, section “Data collection” |
| Field notes | 20 | Were field notes made during and/or after the interview or focus group? | Not applicable |
| Duration | 21 | What was the duration of the interviews or focus group? | See page 8, section “Sample characteristics” |
| Data saturation | 22 | Was data saturation discussed? | See page 4 and 5, section “Study Setting and Recruitment”” |
| Transcripts returned | 23 | Were transcripts returned to participants for comment and/or correction? | See page 5 and 6, section “Data collection” |
| **Domain 3: analysis and findings** | | | |
| Data analysis | | | |
| Number of data coders | 24 | How many data coders coded the data? | See page 6 and 7, section “Data analysis” |
| Description of the coding tree | 25 | Did authors provide a description of the coding tree? | See page 9 to 15, section “Participants’ perspective on patient-centered communication skills” and table 4 “Main and sub-categories with exemplar quotes” |
| Derivation of themes | 26 | Were themes identified in advance or derived from the data? | See page 6 and 7, section “Data analysis” |
| Software | 27 | What software, if applicable, was used to manage the data? | See page 6 and 7, section “Data analysis” |
| Participant checking | 28 | Did participants provide feedback on the findings? | See page 5 and 6, section “Data collection” |
| Reporting | | | |
| Quotations presented | 29 | Were participant quotations presented to illustrate the themes/findings? Was each quotation identified? e.g. participant number | See page 9 to 10, section “Participants’ perspective on patient-centered communication skills” in table 4 “Main and sub-categories with exemplar quotes” |
| Data and findings consistent | 30 | Was there consistency between the data presented and the findings? | See page 9 to 15, section “Participants’ perspective on patient-centered communication skills” and table 4 “Main and sub-categories with exemplar quotes” |
| Clarity of major themes | 31 | Were major themes clearly presented in the findings? | Not applicable (due to applied analysis method) |
| Clarity of minor themes | 32 | Is there a description of diverse cases or discussion of minor themes? | Not applicable (due to applied analysis method) |

Tong A, Sainsbury P, Craig J. Consolidated criteria for reporting qualitative research (COREQ): a 32-item checklist for interviews and focus groups. International Journal for Quality in Health Care. 2007. Volume 19, Number 6: pp. 349 – 357
