# Supplementary material for: Development of a Patient‐Centered Communication Skills Training: A Qualitative Exploration of Nurse Managers' Perspectives
Source: Nurs Open. 2026 May 21;13(5):e70605. doi: 10.1002/nop2.70605 (PMC13240314; doi:10.1002/nop2.70605)
Supplement: Supplementary file 2 — File S2: List of possible aspects to include in a communication skills training. [file NOP2-13-e70605-s002.docx]

Possible aspects to include in a communication skills training

- Shared decision-making
- Conversation about ending therapy
- Communication on death & dying
- Breaking bad news
- Managing emotions
- Communication with family/relatives
- Communication with „demaning“ patients
- Navigating helplessness
- Communication with patient dissatisfaction (e.g., organizational problems)
- Phone conversations, including with relatives
- Communication after sever diagnosis
- Communication in stressful situations/when time is limited
